# Engineering CAR-T Cells for Improved Function Against Solid Tumors

**DOI:** 10.3389/fimmu.2018.02493

**Published:** 2018-10-29

**Authors:** Michael A. Morgan, Axel Schambach

**Affiliations:** ^1^Hannover Medical School, Institute of Experimental Hematology, Hannover, Germany; ^2^REBIRTH Cluster of Excellence, Hannover Medical School, Hannover, Germany; ^3^Division of Hematology/Oncology Boston Children's Hospital, Harvard Medical School, Boston, MA, United States

**Keywords:** chimeric antigen receptor, tumor, cancer, genetic engineering, immunosuppression

## Abstract

Genetic engineering T cells to create clinically applied chimeric antigen receptor (CAR) T cells has led to improved patient outcomes for some forms of hematopoietic malignancies. While this has inspired the biomedical community to develop similar strategies to treat solid tumor patients, challenges such as the immunosuppressive character of the tumor microenvironment, CAR-T cell persistence and trafficking to the tumor seem to limit CAR-T cell efficacy in solid cancers. This review provides an overview of mechanisms that tumors exploit to evade eradication by CAR-T cells as well as emerging approaches that incorporate genetic engineering technologies to improve CAR-T cell activity against solid tumors.

## Introduction

**Figure 1:**
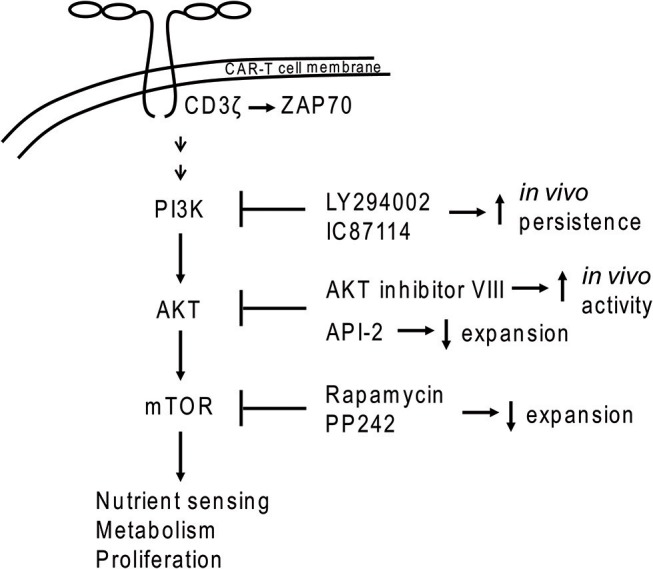
PI3K-AKT-mTOR signaling in CAR-T cells. Dimerization of CAR molecules mimics T cell receptor activation of the PI3K signal transduction cascade. Application of some inhibitors (e.g., PI3K inhibitors LY294002 and IC87114 or the AKT inhibitor VIII) during *ex vivo* expansion led to increased *in vivo* persistence of CAR-T cells.

**Figure 2:**
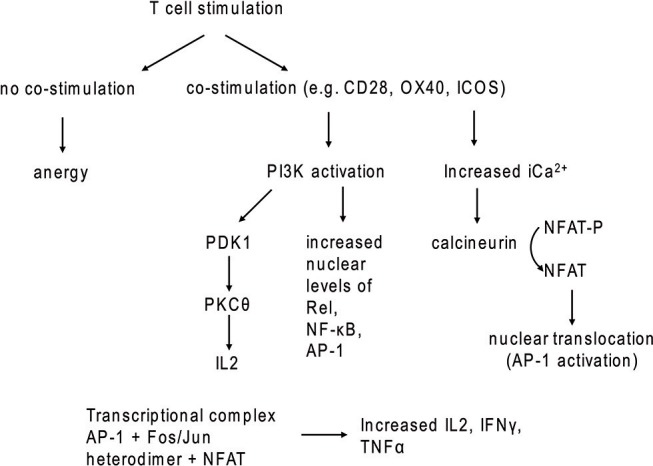
T cell stimulation with and without co-stimulatory signaling. In the absence of co-stimulatory molecules, TCR stimulation leads to anergy.

Reconstitution of effective immune function is a major goal of immunotherapies. In the context of cancer, including solid tumors, the complex interaction of various immune cell sub-populations may have to be re-established to obtain adequate tumor control or eradication. Here, the normal functions of T cells to either regulate immune responses or directly kill infected or cancer cells can be exploited and improved by genetic modification. One of the currently most intensely explored methods to enhance T cell function with the aim to improve cancer patient treatment is the introduction of chimeric antigen receptors (CARs) to generate CAR-T cells with greater anti-tumor activity. CARs are synthetic receptors that contain an antigen recognition domain, e.g., a single chain variable fragment (scFv) that binds to a tumor-associated antigen, a hinge region to provide flexibility to the scFv, a transmembrane domain and a signaling domain with or without co-stimulatory domains that activate the cytotoxic functions of the CAR-T cells upon antigen recognition. For example, most CAR constructs use the CD3ζ signaling chain to stimulate cytotoxic CAR-T activity, which mimics the natural biologic T cell activation pathway, with activation of down-stream signal transduction proteins such as ZAP70, NFAT, and PI3K-AKT-mTOR (Figures 1, 2) (1, 2). Analogous to CD3ζ activation following engagement of the T cell receptor (TCR) in non-modified T cells, activation of CD3ζ signaling in CAR-T cells results in production of cytotoxic cytokines (e.g., IFNγ, TNFα) as well as cytokines to recruit and activate additional immune T cells (e.g., IL-2, IL-10, IL-17) (3–6). In addition to TCR engagement, efficient T cell killing requires simultaneous signaling through a co-stimulatory protein. For example, cross-linking of CD28 or the tumor necrosis factor receptor (TNFR) family members ICOS or 4-1BB results in costimulatory signaling in T cells. Therefore, domains of these natural co-stimulatory proteins are also incorporated in many CAR-T cell designs.

Application of CAR-T cells in some blood malignancies has generated unprecedented responses in B-cell neoplasms, including leukemia and multiple myeloma (7–20). As a result, many resources world-wide are devoted to the development of CAR-T cells to recognize additional tumor-associated antigens or neoantigens to extend this success to treatment of additional cancers, including solid tumors. Engineering approaches to increase CAR-T cells anti-tumor activity, including T cell infiltration into solid tumors, T cell persistence, recruitment/activation of additional anti-tumor immune cells, can exploit mechanisms tumors employ to create an immunosuppressive niche. As discussed below, tumors secrete cytokines to recruit various tumor-associated cells, which, in turn, secrete anti-inflammatory cytokines and/or express ligands for immune checkpoint receptors, which can block CAR-T cells from infiltrating the tumor as well as cause CAR-T cell exhaustion, thus leading to a general decrease in the anti-tumor activity of T and CAR-T cells. This review provides an overview of pro-tumor cell activities in the tumor microenvironment and explores some of the strategies that may help to increase CAR-T cell persistence and functionality with the aim for improved activity against cancer.

## Tumor microenvironment challenges to CAR-T cell function

Tumor cells shape the tumor microenvironment via production and secretion of cytokines that can inhibit T cell function directly or indirectly by recruitment of immunosuppressive cell types (21). Challenges of the tumor microenvironment to T and CAR-T cell activity include hypoxia, metabolic reprogramming conditions, and immunosuppressive signaling through cell checkpoint receptors, all of which serve to protect tumor cells from elimination. As a means of protection of “self,” T cells express inhibitory receptors as a concept called checkpoint inhibition. The most widely studied immune checkpoint receptor-ligand interactions are the programmed cell death 1 (PD1)/programmed cell death ligand 1/2 (PD-L1/2), cytotoxic T-lymphocyte antigen 4 (CTLA4)/CD80/CD86, T-cell immunoglobin and mucin domain 3 (TIM-3)/Galectin-9 and phosphatidylserine on surface of apoptotic cells, and lymphocyte-activated gene-3 (LAG-3) / LSECtin (22, 23). Tumors exploit these immune tolerance signaling pathways to induce T and CAR-T cell exhaustion, which is exhibited by loss of proliferative capacity and decreased production of cytokines such as IL-2, TNF-α, and IFN-γ. Furthermore, exhausted T cells express elevated levels of inhibitory receptors, including PD1, CTLA-4, TIM-3, and LAG-3 and higher expression of these receptors was associated with more advanced disease stage in cutaneous T-cell lymphoma patients (24, 25). TIM-3 expression on tumor infiltrating T cells was predictive for poor outcome in renal cell carcinoma patients (26). In addition to T cells, expression of TIM-3, LAG-3, PD1, and PD-L1 was recently demonstrated on B cells, macrophages, natural killer cells, and dendritic cells in effusions obtained from mesothelioma patients (27). While this study evaluated samples from only a small number of patients (*n* = 6), the observation of exhaustion markers on additional immune cells that interact with T cells in order to orchestrate optimal anti-tumor activity may have important implications for control of solid tumors by CAR-T cells.

Several different cell types (e.g., cancer-associated fibroblasts, regulatory T cells, myeloid-derived suppressor cells, and tumor-associated macrophages) comprise the tumor microenvironment and can inhibit T and CAR-T cell function through distinct and overlapping mechanisms (21, 28–32).

**Figure 3:**
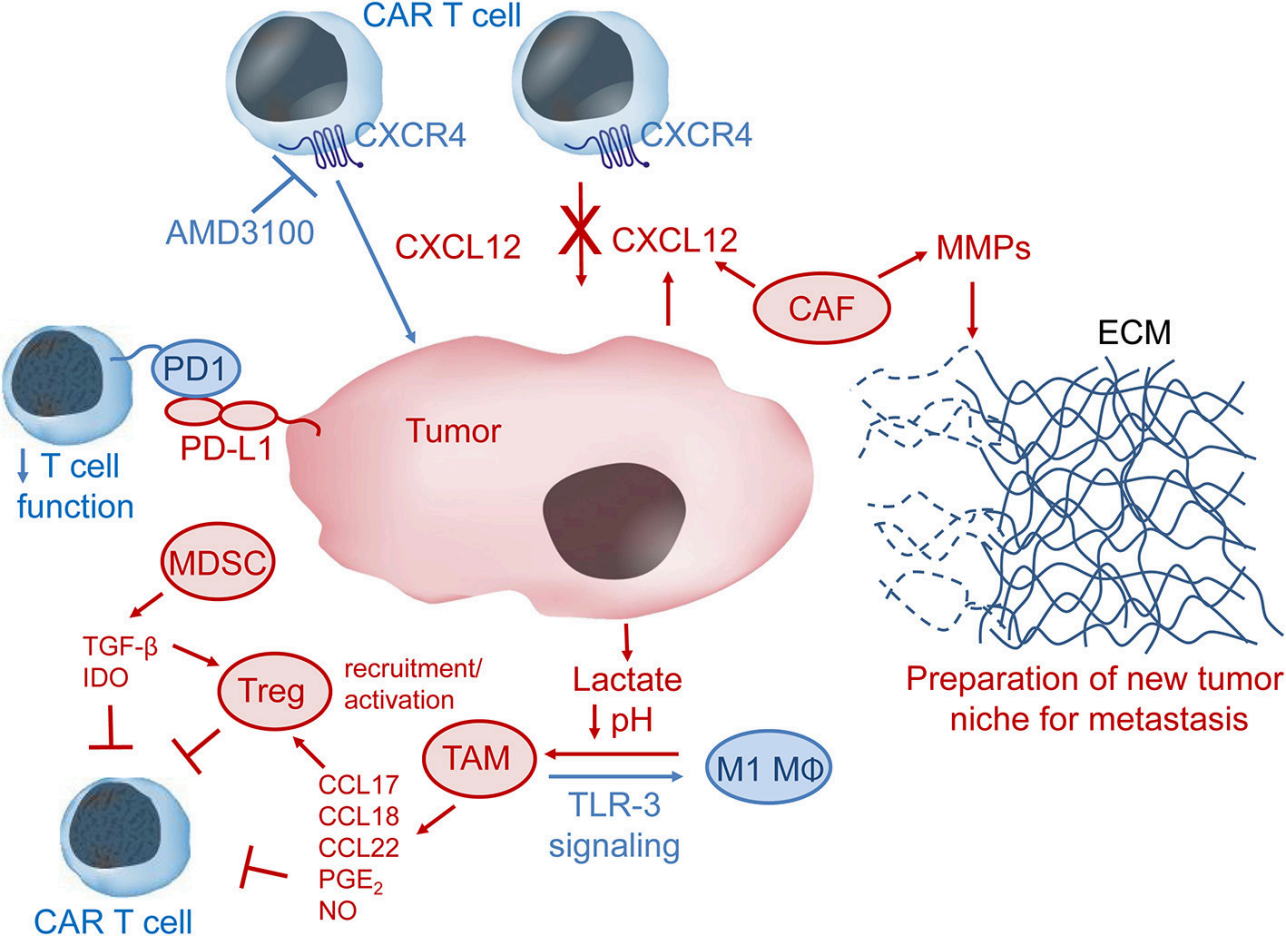
The immunosuppressive tumor microenvironment (TME). CXCL12 in the TME may recruit CXCR4-expressing immunosuppressive cells such as tumor-associated macrophages (TAM), myeloid-derived suppressor cells (MDSC), cancer-associated fibroblasts (CAF), and regulatory T cells (Treg) to the tumor niche. CAF can secrete metallomatrix proteins (MMPs) that lead to remodeling of the extracellular matrix (ECM) via degradation of ECM proteins such as collagen, elastin, fibronectin and laminin. ECM remodeling may be important for tumor invasion and metastasis. Lactate produced by tumor cells leads to lower pH in the TME and can facilitate polarization of M1 macrophages (MΦ) to immunosuppressive TAM, which produce several chemokines and other factors, such as prostaglandin E2 (PGE_2_) and nitric oxide (NO), that inhibit CAR-T cells either directly or via activation of Treg cells. Stimulation of toll-like receptor 3 (TLR-3) signaling may convert TAM to pro-inflammatory M1 MΦ. MDSC produce and secrete the immunomodulatory factors transforming growth factor β (TGF-β) and indoleamine 2,3-dioxygenase (IDO), which inhibit CAR-T cell anti-tumor activity. Immune checkpoint inhibition, e.g., via interaction of programmed cell death 1 (PD1) on T cells with programmed cell death ligand 1 (PD-L1) on tumor or tumor-associated cells, results in down-regulation of T cell activity. Pharmacologic inhibition of the CXCL12 receptor CXCR4 might help CAR-T cells overcome the CXCL12 tumor barrier and thus increase CAR-T cell anti-tumor activity.

Cancer-associated fibroblasts (CAFs) are a major type of stromal cells that occupy the solid tumor microenvironment (33, 34). Activation of fibroblasts by transforming growth factor-β (TGF-β), CXC chemokine ligand 12/stromal cell-derived factor-1 (CXCL12/SDF-1) and IL-6 is common in solid tumors. In contrast to fibroblasts in healthy tissues, CAFs tend to stay in the activated state, through which they may promote tumor metastasis by remodeling the extracellular matrix (ECM) via secretion of matrix metalloproteases (MMP) 2 and 9, which cleave ECM proteins (Figure 3) (28). Tumor microenvironments often contain the chemokine CXCL12 and this was shown to be secreted by CAFs in a murine model of pancreatic ductal adenocarcinoma (30). CAFs were also shown to produce CXCL12 in human breast carcinomas and non-small lung cancer (35, 36). Of clinical interest, CXCL12/CXCR4 levels are increased in many cancers, including breast cancer, pancreatic cancer, oral squamous cell carcinoma, ovarian cancer, cervical carcinoma, and gastric cancer (37–45). CXCL12 may serve to prevent adequate T and CAR-T cell penetration into or recognition of the tumor by forming a barrier of CXCR4^+^ immunosuppressive cells.

Regulatory T (Treg) cells are important for self-tolerance but also contribute to the immune privileged tumor niche by suppression of effector T cell activity. Tregs were shown to home to the bone marrow via CXCL12/CXCR4 signaling in prostate cancer patients with bone metastases (29). The authors postulate that Tregs help create an immunosuppressive niche to aid formation of bone metastases. In an orthotopic mouse model of head and neck squamous cell carcinoma, increased TIM-3 expression was observed on CD8^+^T cells and Tregs after radiation therapy and PD-L1 inhibition (46). Addition of anti-TIM-3 antibodies to the treatment strategy resulted in increased T cell cytotoxicity and improved survival, but the tumors still relapsed. Depletion of Tregs with an anti-CD25 antibody finally led to rejection of established tumors, presumably by restoration of anti-tumor immunity (46).

Myeloid-derived suppressor cells (MDSCs) inhibit anti-tumor immune function by stimulating the activity of immunosuppressive Tregs, producing reactive oxygen species (ROS) and by secreting anti-inflammatory cytokines like IL-10 and TGF-β. CAR-T cells engineered to express catalase maintained anti-tumor activity in the presence of high H_2_O_2_ levels and also protected NK cell activity from oxidative stress (31). Blockade of immunosuppressive TGF-β signaling via expression of a dominant negative TGF-β receptor II in CAR-T cells improved CAR-T cell proliferation, cytokine secretion, *in vivo* persistence and tumor control in mouse models of human pancreatic cancer (47). MDSC also express PD-L1, which can cause T and CAR-T cell exhaustion by binding to PD1. Expression of indoleamine-2, 3-dioxygenase (IDO) by MDSCs can also lead to T and CAR-T cell anergy (32). In a murine tumor model, the immune suppressive function of MDSCs was determined to be a result of metabolic reprogramming, where MDSCs that had the highest suppressive activity also had higher glycolysis levels (48). Inhibition of mTOR with rapamycin led to decreased tumor growth via lower glycolytic and suppressive activity of MDSCs. Similarly to CAFs, MDSCs may also be important for tumor metastasis by remodeling the extracellular matrix via production of MMP9 (49).

Tumor-associated macrophages (TAMs) are another type of immune suppressor cells that inhibit T and CAR-T cell function in the tumor niche. Lactate generated by tumor cells as a by-product of aerobic glycolysis can cause polarization of cytotoxic and inflammatory M1 macrophages to TAMs, which are immunosuppressive macrophages that exhibit an M2 phenotype (50). TAMs can recruit Tregs to the tumor microenvironment via secretion of chemokines (e.g., CCL17, CCL18, CCL22) or even induce Treg suppressor activity by secretion of prostaglandin E2 and IL-10 (21). In addition to production and secretion of factors to recruit and stimulate Treg activity, TAMs can also contribute to protection of tumors from eradication by T and CAR-T cells through expression of PD1 ligands PD-L1/PD-L2 or expression of TIM-3 (51, 52). TAM production of nitric oxide was also shown to contribute to resistance against cisplatin, which is commonly used to treat solid tumors (53). Furthermore, resident macrophages were shown to be important mediators of cytokine release syndrome, a sometimes fatal adverse event that limits CAR-T cell therapy, via production of IL-6, IL-1 and nitric oxide (54).

In summary, the immunosuppressive activity of these various tumor-associated cells negatively impact CAR-T cell persistence, penetration into the tumor and overall anti-tumor activity. Possible strategies to overcome these obstacles will be explored in the following paragraphs.

## Choice of T cell population for improved CAR-T cell function

*In vivo* persistence of CAR-T cells may be a result of the T cell population(s) selected for CAR modification as well as *ex vivo* CAR-T cell expansion procedures. T cells can be divided into several subsets, including naïve T cells (T_N_) (CD45RA^+^CCR7^+^), central memory T cells (T_CM_) (CD45RA^−^CCR7^+^), effector memory T cells (T_EM_) (CD45RA^−^CCR7^−^), effector T cells (T_EFF_) (CD45RA^+^, CD45RO^−^ CCR7^−^ CCR7^−^CD57^+^) and stem cell memory T cells (T_SCM_) (CD62L^+^CCR7^+^CD45RA^+^CD45RO^−^CD95^+^) (55, 56). Increased anti-tumor activity might be achieved if the optimal T cell populations can be identified to generate CAR-T cells and may even allow decreased CAR-T cell doses for each treatment (57). T cell populations currently used for generation of clinical CAR-T cells often include unselected PBMC. However, high inter-patient variation of T cell function and maturation may contribute to the variable success rates in some settings. Attempts to standardize CAR-T cell production include isolation of T cell subpopulations, such as CD4^+^, CD8^+^, CD62L^+^ (to have high numbers of naïve and T_CM_) (58). Of interest, CD8^+^ T_SCM_ were recently used to produce clinical grade CD19-specific CAR-T cells to be tested in a phase 1 trial in patients with B-cell malignancies (59). Another recent study achieved balanced CD4/CD8 ratios with 50% T_CM_ and 46% T_SCM_ from small amounts of blood (60).

It was recently demonstrated that lack of CAR-T cell *in vivo* persistence was due to a lower percentage of naïve T cells (T_N_) vs. effector memory T cells (T_EM_) prior to *in vivo* delivery as the proportions of the T cell subpopulations became skewed during *ex vivo* cultivation and expansion (61). It may be important to standardize T cell isolation, *ex vivo* cultivation and expansion in order to achieve robust comparability among studies.

Earlier work showed that the T_EM_ population can be supported by inhibition of the PI3K/AKT/mTOR pathway (62, 63). PI3K inhibition by LY294002 or IC87114 during *ex vivo* expansion improved CAR-T cell *in vivo* persistence without impacting CAR-T cell yield (61). The same study demonstrated that use of inhibitors against AKT (API-2), mTOR (rapamycin, PP242 = Torkinib) or glycolysis (DCA = dichloroacetate) led to decreased numbers of CAR-T cells. In contrast, another study demonstrated that use of AKT inhibitors (Akti-1/2 or AKT inhibitor VIII) during *ex vivo* expansion generated CAR-T cells with greater activity in a CD19^+^ mouse tumor model without negatively impacting CAR-T cell expansion (64). Differences between these two studies may be due to the distinct properties of the AKT inhibitors employed. Although these concepts were largely developed in the setting of hematopoietic malignancies, they could also impact the effectiveness of CAR-T cell strategies to treat solid tumors.

## Combination therapies to augment CAR-T cell anti-tumor activity

One interesting strategy to overcome immune suppression and generate a more robust antitumor immune response is to combine cancer cell specific CAR constructs and monoclonal antibodies that disrupt checkpoint inhibition (e.g., anti-PD1, anti-PD-L1, anti-TIM-3, anti-LAG-3 antibodies) (65). Currently, monoclonal antibody-based high affinity checkpoint inhibitors are tested against PD1 (nivolumab; lambrolizumab; pidilizumab, pembrolizimab, MGD013), LAG-3 (MGD013), and TIM-3 (lirilumab) to support anti-tumor activity of T cells (66–68).

As discussed above, PD1 expression can lead to T cell exhaustion in which the T cell effector functions are compromised and PD1 inhibitors may re-establish the anti-tumor responses by preventing T cell exhaustion. Ibrutinib, a Bruton's tyrosine kinase inhibitor, was shown to down-regulate PD-L1 expression on tumor cells and PD1 in CD4^+^ and CD8^+^ T cells via inhibition of STAT3 and also decreased IL-10 production by chronic lymphocytic leukemia (CLL) cells in patients (69). PD1 and CTLA-4 ligand binding were shown to decrease glucose metabolism and inhibit AKT activation. CTLA-4 blocked AKT activation via activated protein phosphatase 2A and PD1 inhibited AKT activation by blocking PI3K activation through CD28 (70). Thus, while inhibition of PI3K activity during *ex vivo* CAR-T cell expansion may improve persistence of the final CAR-T cell product, *in vivo* PI3K inhibition has deleterious effects on CAR-T cell function.

Conversion of pro-tumor TAMs to anti-tumor macrophages (e.g., M1 macrophages) was accomplished *in vitro* and in an *in vivo* murine tumor model via stimulation of toll-like receptor-3 (TLR-3) signaling by administration of poly (I:C) (71). TLR-3 stimulation caused functional changes in TAMs, including increased phagocytic activity and upregulation of CD80 and CD86 expression with subsequent induction of CD4 T cell proliferation and tumor regression (71). The authors postulate that interferon-αβ (IFN-αβ) signaling may control the TLR-3 ligand-induced reversion of TAMs to M1 macrophages as application of anti-IFN-αβ blocking antibodies led to tumor progression even in TLR-3 ligand treated mice. As TAMs are thought to inhibit T and CAR-T cell activity, co-administration of molecules that can diminish the amount of TAMs in solid tumors may improve the efficacy of immunotherapeutic strategies in the setting of solid tumors.

In a murine model of glioblastoma multiforme that expressed epidermal growth factor receptor variant III, the thalidomide-based drug lenalidomide improved *in vivo* CAR-T cell function (lentiviral vector—anti-EGFRvIII-scFv, CD28, 4-1BB, and CD3ζ) with improved proliferation, persistence, and formation of immunological synapses (72). Chronic lymphocytic leukemia (CLL) cells produce IL-10 via STAT3 signaling, which can suppress T cell effector function. Lenalidomide was recently demonstrated to inhibit STAT3 phosphorylation, and thus IL-10 production, in CLL cells by blocking CXCL12-CXCR4-IL-10-STAT3 signal transduction, which reversed suppression of T cell effector function (73). As STAT3 phosphorylation via the CXCL12-CXCR4 signaling axis was also demonstrated in solid tumors, including bladder cancer, breast cancer, and small cell lung cancer, inclusion of the immunomodulatory drug lenalidomide may help increase CAR-T cell function in these settings.

CXCL12 inhibits T cell trafficking into tumors by binding the T cell surface receptor CXCR4 and pharmacologic inhibition of CXCR4 with AMD3100 (plerixafor), a drug used for mobilization of hematopoietic stem cells from bone marrow, led to increased T cell infiltration into tumors and synergistically decreased cancer cell numbers when combined with anti-PD-L1 therapy (30). For example, CXCR4 inhibition with AMD3100 treatment led to increased T cell-mediated antitumor activity with concomitant reduction of Tregs, which resulted in improved survival in ovarian cancer and melanoma immunocompetent mouse models (74). Additional evidence that AMD3100 modulates immunosuppression in solid tumors was recently demonstrated in orthotopic mouse models of malignant mesothelioma (75). The authors reported significantly improved tumor control with a combinatorial therapeutic approach that included simultaneous application of AMD3100 and an immune-activating fusion protein that targets mesothelin, which is expressed on mesothelioma. Decreased Treg infiltration in tumors showed that AMD3100 lowered PD1 expression on CD8^+^ T cells and converted Tregs into helper-like cells (CD4^+^CD25^−^Foxp3^+^IL2^+^CD40L^+^). While incorporation of AMD3100 into CAR-T cell treatment regimens has yet to be reported, use of AMD3100 to block the CXCL12-CXCR4 signaling axis was found to promote PD1 inhibition in hepatocellular carcinoma and pancreatic cancer (30, 76). Furthermore, inhibition of CXCL12 with the L-RNA-aptamer NOX-A12, which impedes CXCL12 interaction with CXCR4 and CXCR7, led to greater tumor infiltration by T and natural killer (NK) cells with an improved anti-PD1 therapy in a mouse model of colorectal cancer (77).

All-trans retinoic acid (ATRA), a drug commonly used to release the differentiation blockade in acute promyelocytic leukemia, was found to improve the anti-sarcoma activity of a third generation CAR (14g2a scFv, CD28, OX40, and CD3ζ) designed to target GD2^+^ cells by almost complete elimination of myeloid-derived suppressor cells (MDSC) (78). Similarly, application of the tyrosine kinase inhibitor (TKI) sorafenib increased CD8^+^ T cell trafficking to tumors as well as anti-tumor activity in a murine tumor model (79). These beneficial effects were at least partially due to sorafenib-induced decrease of MDSC and Tregs in the TME. Thus, sorafenib treatment may also lead to improved activity of CAR-T cells against solid tumors.

Oncoproteins expressed in tumor cells, such as mutant EGFR in non-small cell lung cancer cells (NSCLC), can promote higher expression levels of PD-L1 (80). EGFR inhibition with the TKI gefitinib led to decreased PD-L1 expression in NSCLC tumor cell line models and EGFR-induced PD-L1 expression was shown to be dependent upon ERK1/2 signaling down-stream of EGFR as treatment with the ERK1/2 inhibitor SCH772984 also led to diminished PD-L1 expression (81). A high level of T cell apoptosis was observed upon co-cultivation with PD-L1 expressing tumor cells, and T cell apoptosis was blocked by addition of an anti-PD1 antibody or gefitinib, suggesting that combining targeted TKI therapy with CAR-T cells may increase the persistence of CAR-T cells (81). As an additional indicator for T cell fitness, increased IFNγ production was observed in co-cultivation experiments that included gefitinib. Treatment with the EGFR inhibitor erlotinib also led to decreased PD-L1 expression in tumor cell lines that harbor mutant EGFR but not in cells that have wild-type EGFR, further supporting the link between activated EGFR signaling and PD-L1 expression (82). These authors also performed multivariate analysis of 164 NSCLC patients to compare the tumor pathologic stage (IA, IB, IIA, IIB, IIIA, IIIB), patient age, sex, smoking status, histology (adenocarcinoma vs. squamous cell carcinoma) and *EGFR* status and found *EGFR* mutations and histology to be independent variables for high PD-L1 expression (*P* = 0.027 and *P* = 0.046, respectively).

## Novel epigenetic approaches to improve CAR-T cell function

As discussed above, tumors and tumor-associated cells can inhibit T and CAR-T cell anti-tumor activity via PD1- PD-L1/L2 signaling. Thus, control of PD1 expression on T/CAR-T cells provides an opportunity for T/CAR-T cells to overcome this inhibitory effect. Gene expression can be modulated via factors that modify chromatin structure, such as histone acetyltransferases and histone deacetylases (HDAC), with open chromatin structures (i.e., containing acetylated histones) available for gene transcription and “closed” chromatin structures (i.e., regions with hypoacetylated histones) as epigenetically silenced genomic regions. Satb1 (Special AT-rich binding protein 1) recruits HDAC1, which leads to transcriptional repression (83). Satb1 was also shown to be important for regulation of PD1 expression and T cell anti-tumor activity (84). Satb1 expression induced by TCR and costimulatory signals inhibited PD1 expression. TGF-β, an immunoregulatory cytokine commonly present in the tumor microenvironment, led to reduced Satb1 expression in T cells and concomitant increase in PD1 expression (84). These experiments were accomplished in unmodified T cells, thus exploration of the role(s), including possible contribution to tonic signaling, Satb1 or other epigenetic modulators may have in CAR-T cells might reveal novel strategies to increase CAR-T cell effectiveness against solid tumors.

The potential for epigenetic modification strategies to improve anti-tumor activity of CAR-T cells is supported by additional studies. The histone deacetylase inhibitor ACY241 was found to reduce tumor cells, Tregs and MDSC as well as PD1 expression on CD8^+^T cells (85). The functional activity of adoptive T cells in a melanoma tumor model was improved by co-treatment with the histone deacetylase inhibitor LAQ824 (86).

## Effects of vector and CAR designs on CAR-T cell function

In addition to optimizing *ex vivo* CAR-T cell expansion protocols, the choice of a suitable and tailored vector system to deliver improved CAR constructs to T cells may also be important for CAR-T cell anti-tumor activity. In this section, we will thus review different vector and CAR designs and architectures.

As described above, CARs are synthetic receptors composed of a tumor antigen binding domain and an intracelluar CD3ζ-derived effector domain. Accordingly, all components of next generation CARs need to be carefully chosen: **(I)** the scFv or an alternative ligand that ideally exhibits specific targeting with a reasonable on-tumor and low off-target activity; **(II)** the spacer and hinge regions can also impact the antibody/ligand binding avidity and three-dimensional access to tumor antigens; **(III)** the choice of the components of intracellular effector domain is critical to mediate balanced CD3 signaling and T cell persistence; and **(IV)** the combination of I, II and III need to be rationally chosen and experimentally tested for each tumor and tumor antigen, respectively. The features important for optimization of scFv are extensively reviewed elsewhere, thus our discussion will continue with choice of hinge and spacer regions.

The extracellular hinge and spacer component may also influence CAR-T cell persistence and function. Comparison of short (12 amino acids) and long (IgG4 hinge-CH2-CH3 sequence, 229 amino acids) spacers in a murine model demonstrated superior *in vivo* expansion of CAR-T cells containing short spacers (87). The CAR-T cells outfitted with the long extracellular spacer/hinge sequence were depleted *in vivo* via activation-induced cell death independent of tumor antigen recognition and the scFv, mediated by FcγR in the CH2 sequence. Its deletion resulted in CAR-T cells with improved *in vivo* persistence and anti-tumor function (87). This is in line with another report that showed molecular refinements to the CAR spacer could impact multiple biological processes in a solid tumor model, including tonic signaling, cell aging, tumor localization, antigen recognition and superior *in vivo* antitumor activity (88).

In addition, enhanced CAR-T cell functionality was found by ICOS and 4-1BB costimulation, which mediated better functionality and *in vivo* persistence in solid tumor models than 4-1BB CARs (89). However, this depended on design of CAR with the best configuration, i.e., having the ICOS transmembrane domain linked to the ICOS intracellular signaling domain followed by the 4-1BB and CD3ζ domains.

As another important variable, tailored and fine-tuned dosing of the CAR expression is necessary, which is interlinked with the choice of an appropriately designed vector and the number of integrated vector copies. Gammaretro- and lentiviral vectors as well as Sleeping Beauty transposon based vectors are frequently used for this purpose (7, 9, 15, 19, 90). Long terminal repeat (LTR)-driven gammaretroviral vectors exploit strong and compact retroviral promoters within the LTRs, which—in conjunction with a retroviral intron—confer high expression levels in human T cells (91). However, depending on the context, even less expression might be more appropriate. Here interestingly, a self-inactivating (SIN) lentiviral vector outperformed an LTR-driven gammaretroviral vector due to better control and lower CAR expression. Noteworthy, the introduction of an internal ribosomal entry site (IRES) to reduce CAR expression from the LTR-driven gammaretroviral vector also lowered tonic signaling and ligand-independent phosphorylation of the CAR-CD3ζ chain and improved CAR-T cell expansion as compared to CAR-T cells not containing the IRES element (92). CAR-T cell exhaustion can also result from tonic activation of the CAR CD3ζ chain due to clustering of CAR scFv independently of antigen recognition and CAR-T cell exhaustion was found to be increased in CARs that contained the CD28 endodomain as compared to those with the 4-1BB endodomain (89, 93). Interestingly, genetic engineering the 4-1BB CAR to disrupt TRAF2 signaling by mutation of TRAF2 binding sites reduced apoptosis and improved proliferation of these CAR-T cells (92).

To further fine-tune CAR expression to a desired level, the following promoters are frequently used to mediate high (e.g., spleen focus forming virus (SFFV) U3, myeloproliferative sarcoma virus (MPSV) U3) and moderate expression levels (e.g., phosphoglycerokinase (PGK), and elongation factor 1a (EF1a) as house-keeping enzyme promoters) (94–96). In addition to transcriptional control, posttranscriptional regulatory motifs can be included to optimize CAR expression, e.g, miRNA sponges, which act on the posttranscriptional level, can be used to de-target expression from specific cell types and lymphoid subcompartments (97).

A split CAR design was recently described in which the CAR is divided into two sequences, one in which the scFv, transmembrane and co-stimulatory domains are attached to a dimerizer domain and a second that contains the CD3ζ signaling domain attached to a dimerizer domain (98). The CAR is only activated when the CAR binds its antigen and when the small molecule dimerizer is present. This work demonstrated exquisite pharmacologic control of CAR-T cell activity and may increase safety as the activity can be turned on at a specific time, for a set duration, and possibly even the site of action can be controlled (98).

Genetic modification of CAR-T cells to inhibit protein kinase A (PKA) localization to the immune synapse led to improved CAR-T cell trafficking into solid tumors (99). Activation of PKA by prostaglandin E_2_ (PGE_2_) and adenosine lead to inhibition of the T cell receptor. Expression of a peptide called “regulatory subunit I anchoring disruptor” (RIAD) in CAR-T cells inhibited PKA association with ezrin, and increased the *in vitro* anti-tumor activity of modified CAR-T cells even in the presence of inhibitory molecules such as PGE_2_ and adenosine (99). The authors also demonstrated increased tumor-infiltrating capacity and enhanced anti-tumor activity of the RIAD-expressing CAR-T cells in a mesothelin-expressing mouse tumor model.

## Novel gene editing approaches to improve CAR-T cell function

In addition to integrating vector systems, novel gene editing tools enriched CAR-T cell strategies to improve their functionality and versatility. Here, the delivery of designer nucleases, such as zinc finger nucleases, TALENs, megaTALs and CRISPR-Cas9, can be utilized to knock out undesired properties and—in the presence of a carefully designed donor template—to knock in genetic information into so-called “safe harbors” or—by combining both strategies—into the TCR locus. Especially, the latter strategy by knocking out the T cell receptor alpha constant (TRAC) locus has the potential to create off-the-shelf CAR-T cells. Combining this with incorporation of HLA-E, a small and relatively conserved HLA, the NK cell response can be prevented, thus creating universally applicable CAR-T cells. Noteworthy, the so generated designer nuclease-treated TCR-negative CAR-T cells have similar anti-tumor activity as CAR-T cells generated by semi-random lentiviral integration (100). Moreover, by deletion of TRAC and simultaneous incorporation of the CAR at one locus, transgene copy number is controlled and the risk of insertional mutagenesis is potentially lower than that for randomly/semi-randomly integrating viral vectors (100). In a recent bridge to transplantation approach in two infant B-ALL patients, use of TALENs to generate universal CAR-T cells (UCART) by knockout of TRAC coupled with CD52 knockout to endow resistance to the monoclonal antibody Alemtuzumab (Campath), which is used to eliminate CD52^+^ lymphocytes, was shown to be feasible (101).

Further exploitation of genome editing technologies to improve CAR-T cell anti-tumor activity include knockout of PD1, CTLA-4, TIM-3, and LAG-3. For example, CRISPR-Cas9 was used to attempt generation of universal CAR-T cells with PD1 and CTLA-4 double knockouts (102).

As described above, the CXCL12-CXCR4 signaling axis seems to play important roles in formation and maintenance of the tumor niche. The CXCR4 receptor on T cells is also a coreceptor for HIV entry and CRISPR-Cas9 gene editing/disruption of CXCR4 conferred CD4^+^ T cell resistance to HIV-1 infection (103). In another approach, electroporation of Cas9:single guide RNA ribonucleoproteins (Cas9RNP) designed to target CXCR4 resulted in loss of high CXCR4 surface expression in about 40% of cells, and these cells could be further enriched by sorting (104).

## Emerging approaches to overcome tumor and milieu immunosuppression

The fourth generation of CAR-T cells is known as T-cells redirected for universal cytokine-mediated killing (TRUCKs) (105, 106). This strategy is based on the knowledge that T cell functions and those of cooperating anti-tumor immune cells can be modulated by several cytokines. As some of these cytokines may exhibit systemic toxicity, the inherent CAR-T cell mechanism of action allows localized delivery of potentially dangerous cytokines. Cytokine expression occurs via NFAT signaling upon antigen recognition by the CAR. In their earlier work, Abken and colleagues demonstrated increased anti-tumor efficiency using TRUCKs to deliver IL-12 to the tumor niche. Improved tumor control occurred due to recruitment of anti-tumor macrophages via IL-12 expression (105, 106). This principle can be extrapolated to other cytokines. Interestingly, IL-18 was found to increase human T cell engraftment and persistence in murine xenograft models, while negatively affecting Treg engraftment and suppressive effects (107). Improved tumor control in murine models of leukemia and melanoma were observed employing a CD19 CAR-T cell construct designed to constitutively co-express IL-18 (108). Using the TRUCK strategy to deliver IL-18 resulted in greater anti-tumor activity of CAR-T cells directed against the carcinoembryonic antigen in a pancreatic tumor model (109).

Another strategy that may be useful to improve CAR-T cell anti-tumor activity is implementation of switch receptors that convert pro-tumor into anti-tumor signals. In this regard, transfer of a PD1-CD28 receptor containing a truncated extracellular domain of PD1 and the transmembrane and cytoplasmic signaling domains of CD28 into CAR-T cells resulted in increased CAR-T cell anti-tumor activity and is a promising concept for future clinical investigation (110).

## Conclusion and outlook

Improved understanding of the complex interactions that occur in the solid tumor microenvironment will lead to improved tailored genetic engineering approaches. For example, exploitation of pro-tumor signaling such as the CXCL12-CXCR4 axis may lead to development of CAR-T cells with navigation systems, exhibiting improved homing to and penetration into solid tumors. A critical point may be the choice of the T cell population selected for CAR-T cell production. Here, novel insight into cell and stem cell biology will guide educated decisions with regard to the choice of the optimal T cell population. As monotherapeutic approaches are seldom effective in tumor control, it may be necessary to target multiple antigens or to explore novel combinations of CAR-T cells and other therapeutic modalities, such as standard chemotherapy and/or radiation therapy, tyrosine kinase inhibitors, epigenetic modulators or other small molecule drugs. This will form a potent arsenal of next generation CAR-T cell strategies to attack solid tumors.

## Author contributions

All authors listed have made a substantial, direct and intellectual contribution to the work, and approved it for publication.

### Conflict of interest statement

AS is co-inventor on a patent application describing alpharetroviral SIN vectors. The remaining author declares that the research was conducted in the absence of any commercial or financial relationships that could be construed as a potential conflict of interest.
